# Is Visual Vertical Perception Altered in Children and Adolescents with Spinal Misalignment? A Cross-Sectional Study

**DOI:** 10.3390/diagnostics16152461

**Published:** 2026-08-04

**Authors:** Alessandro Picelli, Nicola Turri, Rita Di Censo, Irene Chignola, Antonella Dell’Orco, Ilaria Di Maria, Gaspare Crimi, Mirko Filippetti, Nicola Smania, Valentina Varalta

**Affiliations:** 1 Department of Neurosciences, Biomedicine and Movement Sciences, University of Verona, 37134 Verona, Italy; nicola.turri@studenti.univr.it (N.T.); ilaria.dimaria@univr.it (I.D.M.); mirko.filippetti@univr.it (M.F.); nicola.smania@univr.it (N.S.); valentina.varalta@univr.it (V.V.); 2 Department of Neurosciences, University Hospital of Verona, 37126 Verona, Italy; rita.dicenso@aovr.veneto.it (R.D.C.); irene.chignola@aovr.veneto.it (I.C.); antonella.dellorco@aovr.veneto.it (A.D.); 3 Recovery and Functional Rehabilitation Unit, Marzana Hospital, 37142 Marzana, Italy; gaspare.crimi@aulss9.veneto.it

**Keywords:** body image, proprioception, scoliosis, spine

## Abstract

**Background/Objectives**. Spinal misalignment in childhood and adolescence may be associated with altered sensory integration and body representation. The subjective visual vertical reflects visual, vestibular, and somatosensory integration, but its relationship with pediatric spinal misalignment remains uncertain. This study investigated the subjective visual vertical using two complementary paradigms. **Methods**. This exploratory, single-center, cross-sectional study included participants aged 7–17 years with clinically identified spinal misalignment. Participants underwent standardized physiatric assessment and subjective visual vertical testing using a luminous-line test and the Bucket Test. Spearman’s rank correlations examined associations with clinical and radiographic variables, with false-discovery-rate control using the Benjamini–Hochberg procedure. Radiographic analyses were restricted to participants with available imaging. **Results**. Fifty-seven participants were included (mean age 12.8 years, SD 2.25), and all completed both tests. Mean subjective visual vertical was 0.053° (SD 0.953) with the luminous-line test and −0.561° (SD 1.165) with the Bucket Test. Radiographs were available for 21 participants. No association remained significant after false-discovery-rate correction. Two luminous-line associations were nominally significant before correction: primary lumbar curve presence (ρ = −0.278, *p* = 0.036) and secondary thoracolumbar curve presence (ρ = −0.324, *p* = 0.015); both had q = 0.721. The tests were moderately correlated (ρ = 0.493, *p* < 0.001). Radiographic sensitivity and subgroup analyses showed no significant findings. **Conclusions**. Subjective visual vertical was not robustly associated with spinal misalignment characteristics in this modest, clinically heterogeneous cohort. Larger controlled studies with standardized imaging and adequately powered severity groups are required.

## 1. Introduction

Spinal misalignment in childhood and adolescence encompasses a spectrum of trunk and spinal alterations, ranging from mild postural asymmetries and non-structural deformities to more clearly defined structural conditions. These abnormalities may involve the coronal, sagittal, and transverse planes and may differ in severity, clinical relevance, and risk of progression during growth. Adolescent idiopathic scoliosis is the most extensively studied condition within this spectrum. Overall, current evidence supports a multifactorial pathogenesis of spinal misalignment, involving genetic and epigenetic mechanisms, altered skeletal growth, hormonal factors, biomechanical factors, and abnormalities in sensorimotor integration. Increasing attention has therefore been directed toward how visual, vestibular, and somatosensory inputs are centrally integrated to maintain postural alignment during growth [1,2,3].

Visual vertical perception is a relevant component of spatial orientation and postural control. The subjective visual vertical is defined as the perceived alignment of a visual stimulus with the true gravitational vertical. Although the subjective visual vertical primarily reflects visual and vestibular processing, it is also influenced by somatosensory information and central multisensory integration. For this reason, subjective visual vertical testing has been used as a clinically accessible method to investigate sensory contributions to balance and upright posture. In rehabilitation research, it is of particular interest because an altered internal reference of verticality may theoretically contribute to persistent postural asymmetry or to difficulty correcting an already established spinal deformity [3].

This hypothesis is particularly relevant in scoliosis, as several studies have suggested that adolescents with idiopathic scoliosis may present disturbances in postural control, body representation, or verticality-related processing. Patients with adolescent idiopathic scoliosis may underestimate trunk deformity or show altered self-perception in relation to the gravitational vertical, supporting the idea that internal spatial representation may be involved in the disorder. Vestibular involvement also remains biologically plausible: a recent systematic review reported recurrent vestibular morphological asymmetries in adolescent idiopathic scoliosis, although the available evidence was insufficient to establish a causal relationship [4,5,6].

However, studies specifically examining the subjective visual vertical in scoliosis have yielded conflicting results. Some authors have reported abnormal subjective visual vertical values in adolescents with idiopathic scoliosis, whereas others have not confirmed a consistent alteration. These discrepancies were reflected in a 2020 systematic review and meta-analysis, which concluded that visual verticality does not appear to be consistently altered in idiopathic scoliosis and emphasized the marked heterogeneity of the available assessment methods and study populations [5]. More recent studies suggest that abnormalities in visual vertical perception may be influenced by associated features such as head position, severity of deformity, or the distinction between bottom-up sensory processing and top-down body-image mechanisms [7,8,9].

The subjective visual vertical can be assessed using different paradigms that vary in their degree of environmental control, equipment requirements, and clinical feasibility. In the present study, a luminous-line test and the Bucket Test were selected as complementary assessment methods. The luminous-line test, performed in a darkened environment with peripheral visual references restricted, was chosen to provide a controlled assessment of perceived gravitational vertical while minimizing the influence of external visual cues [10]. The Bucket Test was selected as a simple, inexpensive, portable, and clinically feasible method in which the bucket itself occludes peripheral visual references [11,12,13]. The simultaneous use of these two paradigms allowed visual vertical perception to be examined both through a more controlled experimental procedure and through a pragmatic clinical tool. It also enabled the consistency of the findings across different testing conditions to be explored, which is particularly relevant given the methodological heterogeneity identified in the previous literature [5].

Accordingly, whether visual vertical perception is altered in children and adolescents with spinal misalignment remains uncertain. Clarifying this issue may help determine whether subjective visual vertical assessment provides clinically meaningful information in the rehabilitation evaluation of young patients with spinal deformities. Therefore, the aim of the present cross-sectional study was to investigate the subjective visual vertical in children and adolescents with spinal misalignment using these two complementary visual paradigms.

## 2. Materials and Methods

### 2.1. Study Design and Reporting Guidelines

This was a single-center, cross-sectional observational study reported in accordance with the Strengthening the Reporting of Observational Studies in Epidemiology (STROBE) statement for cross-sectional studies [14]. The study was conducted in accordance with the Declaration of Helsinki and was approved by the Institutional Review Board of the University of Verona (approval no. 08/18). Written informed consent was obtained from the participants’ parents or legal guardians, and assent was obtained from the participants when appropriate.

### 2.2. Participants

Consecutive children and adolescents aged 7–17 years who underwent evaluation at the scoliosis clinic were considered eligible. For the purposes of this study, spinal misalignment was operationally defined as one or more clinically identified abnormalities involving the coronal, sagittal, or transverse plane. These abnormalities included waist triangle asymmetry, pelvic obliquity, a thoracic, thoracolumbar, or lumbar rib hump, an altered sagittal spinal profile involving thoracic kyphosis or lumbar lordosis, and/or a clinically or radiographically identified thoracic, thoracolumbar, or lumbar spinal curve.

The study was designed to investigate the broader clinical spectrum of spinal misalignment rather than exclusively radiographically confirmed scoliosis; therefore, radiographic confirmation and the availability of a Cobb angle were not eligibility requirements. Exclusion criteria were previous spinal surgery; a history of vestibular disorders; neurological disorders; muscular disorders potentially affecting postural control, spatial orientation, or the variables under investigation; non-correctable visual impairment; and previous exposure to visual vertical testing.

### 2.3. Assessment Procedure and Blinding

The assessment was performed in two separate phases. First, each participant underwent a standardized physiatric evaluation in the scoliosis clinic. Subsequently, subjective visual vertical testing was performed in a different room by a second examiner.

The examiner administering the visual tests was blinded to the severity, anatomical level, and radiographic characteristics of the spinal misalignment. For participants wearing a brace, the examiner was necessarily aware that a spinal condition was present but remained unaware of curve magnitude and anatomical level. Participants were tested while standing without external support. In brace-treated participants, visual vertical testing was performed without the brace.

### 2.4. Clinical and Radiographic Assessment

Demographic and anamnestic data included age, sex, pubertal status, family history of scoliosis, and regular sports participation. Pubertal status was recorded according to the occurrence of menarche in females and voice break in males.

The standardized physiatric examination documented trunk asymmetries, including waist triangle asymmetry and pelvic obliquity, as well as the sagittal spinal profile. Thoracic kyphosis and lumbar lordosis were measured clinically using an inclinometer. Rib hump assessment included its presence, side, anatomical location, and magnitude; magnitude was measured using a scoliometer and recorded in degrees and, when available, in millimeters. The presence, anatomical location, and apparent convexity of clinically identified primary and secondary curve patterns were also recorded.

Radiographic examination was not routinely required for study inclusion because the target population comprised children and adolescents with clinically identified spinal misalignment rather than only patients with radiographically confirmed scoliosis. Spinal radiographs were obtained when clinically indicated to confirm or characterize a suspected structural deformity while avoiding unnecessary exposure to ionizing radiation. When available, radiographic variables included primary and secondary curve magnitude measured using the Cobb method and skeletal maturity assessed using the Risser sign [15,16]. All analyses involving Cobb angles or the Risser sign were restricted to participants with the corresponding radiographic data, and missing radiographic values were not imputed.

### 2.5. Visual Vertical Assessment

Subjective visual vertical was assessed using two complementary visual paradigms: a luminous-line test and the Bucket Test. Both methods quantify the angular deviation between the vertical perceived by the participant and the true gravitational vertical [10,11,12,13]. To minimize potential sequence, learning, and fatigue effects, the order of administration of the two tests was randomized for each participant. For each paradigm, the outcome was expressed in degrees of deviation from the true gravitational vertical. The participant-level score used in the statistical analyses was calculated as the mean angular deviation across the eight trials, with positive and negative values indicating opposite directions of perceived tilt.

#### 2.5.1. Luminous-Line Test

**Figure 1:**
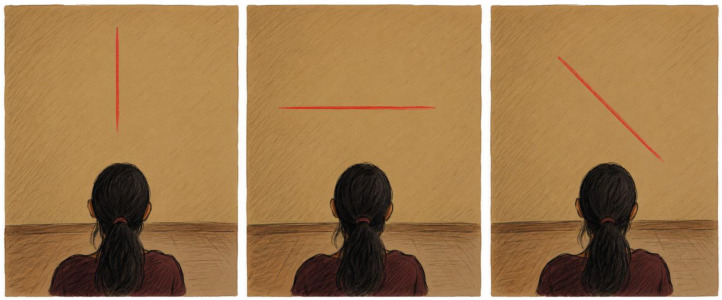
Luminous-line test setup for the assessment of subjective visual vertical.

For the luminous-line test [17], participants stood unsupported in a dark room at a distance of 4 m from the wall. A white line measuring 2.5 m in length and 3 cm in width was projected onto the wall. At the beginning of each trial, the line was displayed in an oblique position to prevent the initial presentation from providing an obvious vertical reference. During each trial, the line rotated at approximately 2°/s in 1° increments. Participants were instructed to verbally indicate when the line appeared to be perfectly aligned with the gravitational vertical, at which point the examiner stopped its movement. Eight trials were performed using the following predefined starting positions: +40°, +20°, −20°, −40°, +20°, −20°, +40°, and −40°. The use of starting positions on both sides of the gravitational vertical was intended to reduce systematic bias related to the initial direction of line rotation. Between trials, participants closed their eyes while the line was returned to the subsequent starting position. After each trial, participants were also asked to rate their confidence in the response. To minimize the influence of external visual references, environmental visual cues were masked, and participants observed the stimulus through a cylindrical tube that restricted the peripheral visual field. The luminous-line test setup and administration procedure are illustrated in Figure 1.

#### 2.5.2. Bucket Test

**Figure 2:**
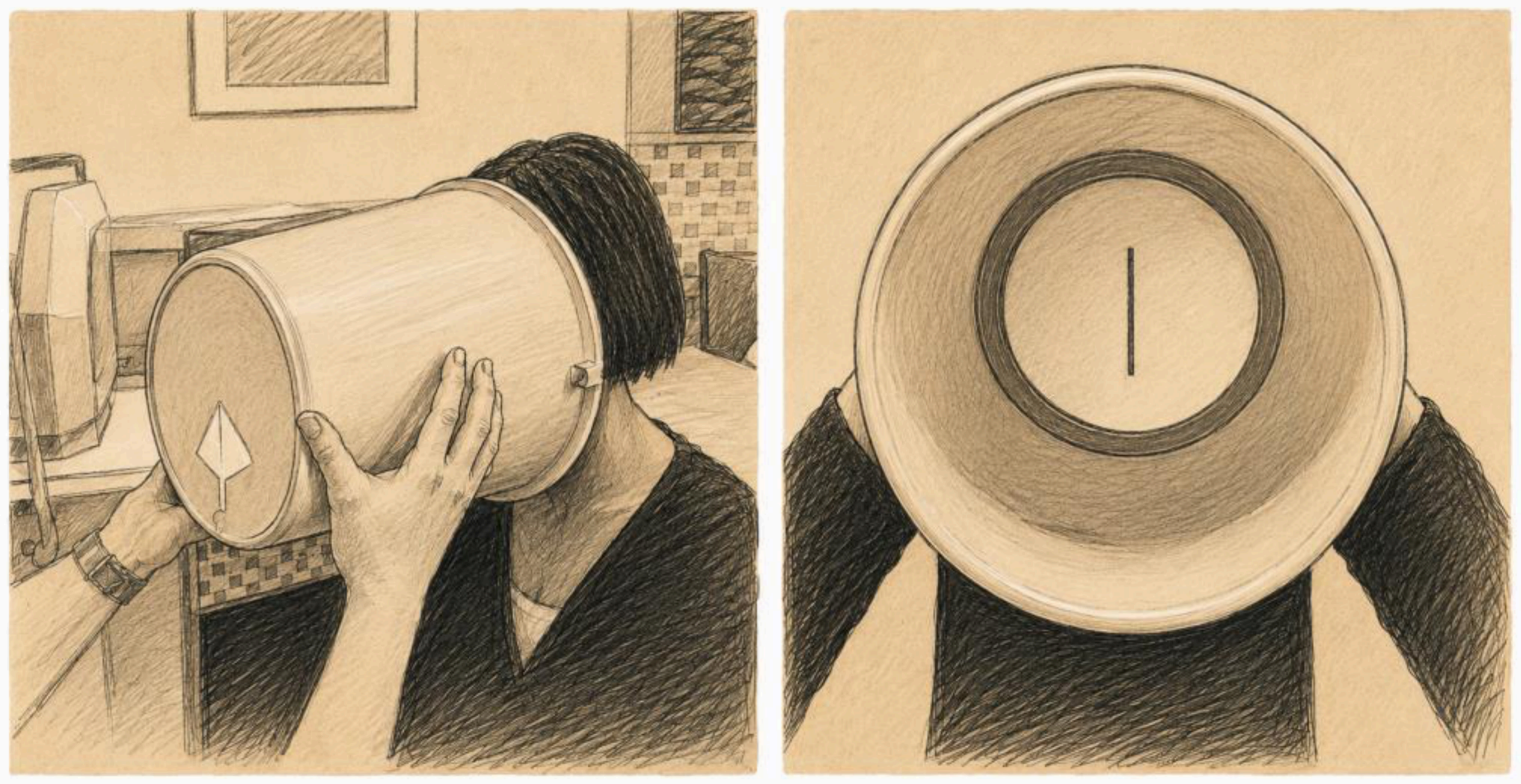
Bucket Test setup for the assessment of subjective visual vertical.

For the Bucket Test, participants looked inside a bucket containing a straight line drawn on its inner base. A protractor and a plumb line positioned on the external base of the bucket allowed the examiner to measure the angular deviation of the internal line from the true gravitational vertical. While standing without external support, participants instructed the examiner to stop rotating the bucket when the internal line appeared to be vertically aligned. Room lighting was maintained to ensure adequate visualization of the line, whereas peripheral environmental references were occluded by the walls of the bucket. Eight measurements were collected for each participant using rotations from both sides of the gravitational vertical. The Bucket Test has been described as a simple, inexpensive, and feasible bedside measure of subjective visual vertical [11,12,13], and its setup is illustrated in Figure 2.

#### 2.5.3. Validity and Reliability of the Subjective Visual Vertical Measures

The luminous-line paradigm performed in darkness is an established laboratory method for assessing perceived gravitational vertical because it minimizes allocentric visual references and permits the angular error relative to the true vertical to be quantified. Its construct and known-groups validity are supported by its ability to detect disturbances of graviceptive processing in neurological populations [10,17].

For the Bucket Test, previous research reported intratest reliability of 92% for both binocular and monocular subjective visual vertical measurements [11]. Reference values and the distribution of Bucket Test measurements have also been investigated in healthy individuals [12]. In addition, its variability and test–retest reliability have been evaluated in comparison with an instrumented virtual subjective visual vertical system [13].

In the present study, eight trials were collected and averaged for each paradigm to reduce random measurement error and the possible influence of the starting position. The correlation between the luminous-line test and the Bucket Test was also examined as an indicator of convergent validity. Nevertheless, the available psychometric evidence refers mainly to adult neurological, vestibular, or healthy populations, and evidence relating specifically to the exact projected-line configuration used in this study and to pediatric populations with spinal misalignment remains limited. The two paradigms were therefore considered complementary rather than interchangeable measures of visual vertical perception.

### 2.6. Statistical Analysis

Statistical analysis was performed using SPSS version 27.0 for Macintosh. The distributions of continuous variables were assessed using the Shapiro–Wilk test. Categorical variables were not subjected to normality testing because the assumption of normality is not applicable to categorical data. Continuous variables were summarized using the mean and standard deviation, the median and first and third quartiles, and minimum and maximum values, as appropriate; categorical variables were summarized using absolute frequencies. Both subjective visual vertical outcomes showed significant departures from normality. Therefore, Spearman’s rank correlation coefficients were used to examine associations between the luminous-line test or the Bucket Test and the clinical, curve-pattern, and radiographic variables. The same rank-based approach was used for ordinal and dichotomous variables to ensure consistency across the exploratory analyses. Given the number of associations examined, the false discovery rate was controlled using the Benjamini–Hochberg procedure across the 54 exploratory correlation tests. Both unadjusted *p*-values and false-discovery-rate-adjusted q-values were calculated and reported. Statistical significance for these analyses was defined as q < 0.05; associations with an unadjusted *p* < 0.05 but q ≥ 0.05 were considered nominally significant and interpreted as exploratory. The correlation between the luminous-line test and the Bucket Test was considered a separate, prespecified convergent-validity analysis and was not included in the multiple-comparison family. Analyses involving radiographic variables were restricted to participants with the corresponding data, and missing radiographic values were not imputed. To examine the consistency of the findings within the radiographically characterized subgroup, a sensitivity analysis was conducted in the 21 participants with available spinal radiographs. Associations with primary Cobb angle were examined in this subgroup, whereas analyses involving secondary Cobb angle and Risser sign were restricted to participants for whom these measures were available. Exploratory post hoc subgroup analyses compared participants with a primary Cobb angle below versus at least 10°, below versus at least 20°, and Risser grade 0–2 versus 3–5. Because the subjective visual vertical outcomes were non-normally distributed and the subgroups were small, comparisons were performed using the Mann–Whitney U test. The six subgroup comparisons were adjusted separately using the Benjamini–Hochberg procedure, with statistical significance defined as q < 0.05. All tests were two-sided.

## 3. Results

Fifty-seven consecutive children and adolescents with clinically identified spinal misalignment were enrolled. The sample included 29 females and 28 males, with a mean age of 12.8 years (SD 2.25). All participants completed both the luminous-line test and the Bucket Test. At the time of assessment, 2 participants (3.5%) were receiving Chêneau brace treatment combined with exercise therapy, 34 (59.6%) were undergoing exercise therapy without a brace, and 21 (36.8%) were under clinical follow-up only. In the two brace-treated participants, subjective visual vertical testing was performed without the brace. Baseline demographic, treatment-related, clinical, and radiographic characteristics are summarized in Table 1.

The Shapiro–Wilk test showed significant departures from normality for both the luminous-line test (W = 0.887, *p* < 0.001) and the Bucket Test (W = 0.926, *p* = 0.002); consequently, Spearman’s rank correlations were used. Table 2 presents the associations between subjective visual vertical and the clinical variables. None remained statistically significant after false-discovery-rate correction.

Spinal radiographs were available for 21 of the 57 participants (36.8%); the Risser sign was available for 16 (28.1%), and secondary Cobb angle measurements were available for 8. Analyses involving radiographic variables were restricted to participants with the corresponding data, and missing values were not imputed. Table 3 presents associations with clinically identified curve patterns and radiographic variables, together with the inter-test association. Two luminous-line associations reached nominal significance in the unadjusted analyses: primary lumbar curve presence (ρ = −0.278, *p* = 0.036) and secondary thoracolumbar curve presence (ρ = −0.324, *p* = 0.015). Neither remained significant after Benjamini–Hochberg correction across the 54 exploratory tests (q = 0.721 for both). No significant associations were identified with primary Cobb angle, secondary Cobb angle, or Risser sign. The luminous-line test and the Bucket Test showed a moderate positive correlation (ρ = 0.493, *p* < 0.001).

Sensitivity analyses within the radiographically characterized subgroup were consistent with the main findings. Among the 21 participants with primary Cobb measurements, 10 had an angle below 10° and 11 had an angle of at least 10°; neither the luminous-line test (U = 62.5, *p* = 0.607) nor the Bucket Test (U = 57.5, *p* = 0.882) differed between groups. Seventeen participants had a primary Cobb angle below 20° and four had an angle of at least 20°; again, no differences were found for the luminous-line test (U = 47.5, *p* = 0.224) or the Bucket Test (U = 32.5, *p* = 0.925). Participants with Risser grades 0–2 and 3–5 also did not differ on the luminous-line test (U = 23.5, *p* = 0.677) or the Bucket Test (U = 34.5, *p* = 0.437). None of the six comparisons was significant after false-discovery-rate correction (all q = 0.925). These analyses were exploratory and underpowered: only four participants had a Cobb angle of at least 20°, the maximum angle was 25°, and no severe scoliosis was represented. The absence of subgroup differences should therefore not be interpreted as evidence of equivalence across the full severity spectrum.

## 4. Discussion

The principal finding of the present study was the absence of robust associations between subjective visual vertical and the clinical, curve-pattern, or radiographic characteristics of spinal misalignment in children and adolescents. Two luminous-line correlations reached nominal significance in the unadjusted analyses, but neither survived false-discovery-rate correction; they should therefore not be interpreted as anatomically specific effects. By contrast, the luminous-line test and the Bucket Test showed a moderate positive correlation, indicating that they assess related, although not interchangeable, components of perceived gravitational verticality.

These findings are broadly consistent with the previous literature. The systematic review and meta-analysis by Obrero-Gaitán et al. concluded that idiopathic scoliosis is not consistently associated with impaired visual verticality and highlighted substantial methodological and clinical heterogeneity [5]. Similarly, Le Berre et al. observed altered subjective postural vertical rather than altered subjective visual vertical in adolescents with idiopathic scoliosis, suggesting that verticality-related abnormalities may involve postural or somatosensory processes more than the visual estimate of gravity alone [18].

The absence of a consistent subjective visual vertical abnormality should not, however, be equated with intact sensorimotor integration. Postural orientation depends on the dynamic weighting of visual, vestibular, and somatosensory inputs according to their reliability and the demands of the task. Within this sensorimotor-reweighting framework, an accurate final estimate of visual vertical may reflect compensation rather than completely preserved multisensory processing [3]. Because both tests were performed under relatively stable sensory conditions, they assessed the perceptual outcome but not the relative contribution or dynamic reweighting of individual sensory channels. Sensorimotor reweighting is therefore a plausible interpretation, not a mechanism directly demonstrated by this study.

This framework may help reconcile the present results with reports of altered body schema, body image, subjective postural vertical, head position, and vestibular morphology in adolescents with idiopathic scoliosis [4,6,8,9,18,19,20]. Subjective visual vertical concerns the orientation of an external stimulus relative to gravity, whereas subjective postural vertical, body schema, and body image concern the perceived orientation, configuration, or appearance of the body. A patient may therefore retain an accurate visual estimate of gravity while showing altered trunk representation or atypical weighting of somatosensory and vestibular information [4,9,18].

Previous studies reporting abnormal subjective visual vertical also differed from the present investigation. Čakrt et al. found greater Bucket Test deviations in adolescents with idiopathic scoliosis [7], Kučerová et al. reported differences in subjective visual vertical together with greater coronal head tilt [8], and Antoniadou et al. described abnormal verticality perception consistent with possible vestibular involvement [20]. These studies generally included selected idiopathic scoliosis samples and healthy control groups and, in some cases, assessed head position or vestibular variables. Abnormalities may therefore be more apparent in specific phenotypes, more severe deformities, or multimodal sensory assessments than in a heterogeneous outpatient cohort undergoing static visual vertical testing.

The moderate inter-test correlation is also methodologically informative. Both paradigms required alignment of a visual stimulus with perceived gravity, but they differed in visual context, environmental control, stimulus characteristics, and examiner involvement. The luminous-line test was conducted in darkness with restricted peripheral references, whereas the Bucket Test was performed under room lighting with the surrounding environment occluded by the bucket. Their moderate rather than strong association supports their use as complementary, not interchangeable, measures.

The broad inclusion criteria improved clinical representativeness but reduced internal validity. The cohort ranged from minor postural asymmetries and non-structural alterations to radiographically documented curves, which may have attenuated phenotype-specific effects. Radiographs were available for 21 participants; this limited analyses of structural severity but did not affect completeness of the primary visual vertical outcomes, because all 57 participants completed both tests. Radiographic analyses were restricted to available data without imputation.

Retaining the full clinically defined cohort was consistent with the study objective and also avoided restricting the analysis to a clinically selected imaging subgroup. Because radiographs were obtained according to clinical indication, an analysis confined to imaged participants could be affected by indication bias. Sensitivity analyses in the radiographic subgroup and post hoc comparisons by Cobb angle and Risser grade were consistent with the overall findings, but they were markedly underpowered: only four participants had a Cobb angle of at least 20°, the maximum was 25°, and no severe scoliosis was represented. These analyses do not establish equivalence across severity categories.

Statistical interpretation also requires caution. Fifty-four exploratory associations were examined in a modest sample. The Benjamini–Hochberg procedure reduced the risk of false-positive findings, but it also reduced power. Accordingly, the null and nominal findings should be viewed as exploratory and hypothesis-generating rather than definitive evidence that no association exists.

The limitations of the study should be carefully considered. First, the absence of a healthy control group prevents direct comparison with typically developing peers and precludes conclusions about group-level abnormality. Second, radiographic data were available only for clinically selected subgroups, limiting power for Cobb angle, secondary curve, and skeletal maturity analyses and introducing possible indication bias. Third, the sample was dominated by mild spinal misalignment and did not include severe scoliosis. Fourth, heterogeneity in spinal presentation and current management—two participants received Chêneau bracing plus exercise therapy, 34 received exercise therapy alone, and 21 were under clinical follow-up—may have obscured phenotype- or treatment-specific effects. Fifth, the study assessed only subjective visual vertical; subjective postural vertical, head alignment, body representation, oculomotor behavior, dynamic postural control, and formal vestibular function were not concurrently evaluated [4,6,8,18]. Sixth, sensorimotor reweighting was not directly tested using sensory-conflict or dynamic paradigms. Finally, the cross-sectional design precludes inference about causality, progression, adaptation, or prognostic value.

The study also has several strengths. Consecutive recruitment reflects the specific outpatient setting, all participants completed both visual vertical tests, and testing was generally performed by an examiner blinded to curve severity and anatomical level. Participants were assessed while standing without external support, and randomization of test order reduced sequence, learning, and fatigue effects. The use of two complementary paradigms further allowed the consistency of findings across a controlled laboratory procedure and a feasible bedside method to be examined.

### 4.1. Clinical Implications

From a clinical perspective, the findings do not support the routine use of subjective visual vertical as an isolated diagnostic, anatomical, or severity marker for pediatric spinal misalignment. A normal value should not be taken as proof of intact sensorimotor integration, and an isolated deviation is not specific to a spinal curve. Subjective visual vertical may nevertheless contribute to a broader evaluation in selected patients with abnormal head position, balance dysfunction, vertigo, altered trunk perception, or suspected vestibular involvement.

### 4.2. Future Research

The Bucket Test is inexpensive, portable, and easy to administer [11,12,13], but this study did not establish diagnostic thresholds, sensitivity, specificity, prognostic value, or responsiveness to treatment. Future studies should include matched healthy controls and prospectively defined, adequately powered subgroups spanning non-structural misalignment and mild, moderate, and severe scoliosis. Sensorimotor reweighting should be examined directly using visual-conflict conditions, altered support surfaces, changes in head orientation, dynamic posturography, and formal vestibular testing. Multimodal protocols incorporating subjective postural vertical, body schema, body image, head alignment, oculomotor measures, and vestibular function may identify phenotype-specific abnormalities [4,5,6,7,8,9,18,19,20]. Longitudinal studies are also needed to determine whether sensory weighting precedes deformity, adapts to progression, or changes with bracing or rehabilitation. Confirmatory studies should prespecify primary outcomes, perform sample-size calculations, and control multiple testing. Overall, the present results do not demonstrate a robust relationship between subjective visual vertical and pediatric spinal misalignment characteristics; they instead suggest that relevant dysfunction, where present, may involve compensatory sensory weighting or domains not captured by static subjective visual vertical testing.

## Figures and Tables

**Table 1:** Demographic, treatment-related, clinical, and radiographic characteristics of the sample.

Min–Max	Value	Statistic/Category	*n*	Variable
6.8–17.0	12.8 (2.25)	Mean (SD)	57	Age, years
—	28/29	Male/Female	57	Sex
—	34/23	Absent/Present	57	Menarche or voice break
0.1–2.4	1.14 (0.53)	Mean (SD)	23	Time since menarche/voice break, years
—	24/33	Absent/Present	57	Family history of scoliosis
—	12/45	Absent/Present	57	Regular sports participation
—	2/34/21	Brace + exercise/Exercise/Follow-up	57	Current management
—	25/13/38	Right > left/Left > right/Total	57	Waist triangle asymmetry
—	9/3/12	Right/Left/Total	57	Pelvic tilt
—	30/26/56	Right/Left/Total	57	Primary rib hump side
—	28/18/10	Thoracic/Thoracolumbar/Lumbar	56	Primary rib hump location
1–11	4.4 (1.9)	Mean (SD)	56	Primary rib hump height, degrees
1–14	5.5 (3.0)	Mean (SD)	45	Primary rib hump height, mm
—	36/21	No/Yes	57	Spinal radiographs available
3–25	11.3 (6.42)	Mean (SD)	21	Primary curve magnitude, degrees Cobb
0–5	0 (0–3.75)	Median (Q1–Q3)	16	Risser sign (0–5)
18–62	39.1 (10.1)	Mean (SD)	57	Thoracic kyphosis, degrees
12–60	36.2 (8.9)	Mean (SD)	57	Lumbar lordosis, degrees
−2–2	0.053 (0.953)	Mean (SD)	57	Subjective visual vertical, luminous-line test, degrees
−4–2	−0.561 (1.165)	Mean (SD)	57	Subjective visual vertical, Bucket Test, degrees

Abbreviations: *n*, number of participants with available data; SD, standard deviation; Q1, first quartile; Q3, third quartile.

**Table 2:** Correlations between subjective visual vertical and clinical variables.

*n*	Bucket Test, ρ (*p*; q)	Luminous-Line Test, ρ (*p*; q)	Clinical Variable
57	−0.028 (0.836; 1.000)	−0.104 (0.440; 0.880)	Thoracic kyphosis (inclinometer)
57	−0.038 (0.780; 1.000)	−0.035 (0.795; 1.000)	Lumbar lordosis (inclinometer)
57	0.176 (0.190; 0.798)	0.078 (0.563; 0.921)	Primary thoracic rib hump (presence)
57	−0.104 (0.440; 0.880)	0.038 (0.776; 1.000)	Primary thoracolumbar rib hump (presence)
57	−0.100 (0.458; 0.880)	−0.144 (0.284; 0.864)	Primary lumbar rib hump (presence)
57	0.117 (0.384; 0.864)	−0.004 (0.974; 1.000)	Primary rib hump on right side
57	−0.117 (0.384; 0.864)	0.004 (0.974; 1.000)	Primary rib hump on left side
56	−0.033 (0.808; 1.000)	0.105 (0.443; 0.880)	Primary rib hump height (degrees)
45	0.110 (0.473; 0.880)	0.237 (0.116; 0.721)	Primary rib hump height (mm)
39	0.064 (0.697; 1.000)	0.009 (0.955; 1.000)	Distance of primary hump from midline (cm)
57	−0.093 (0.493; 0.888)	−0.210 (0.117; 0.721)	Secondary rib hump (presence)
17	0.072 (0.785; 1.000)	−0.099 (0.705; 1.000)	Secondary rib hump height (degrees)
11	0.112 (0.743; 1.000)	0.490 (0.126; 0.721)	Secondary rib hump height (mm)
9	0.057 (0.885; 1.000)	−0.068 (0.863; 1.000)	Distance of secondary hump from midline (cm)

Values are Spearman’s ρ, with the unadjusted *p*-value and false-discovery-rate-adjusted q-value shown in parentheses as ρ (*p*; q). Statistical significance was defined as q < 0.05.

**Table 3:** Correlations between subjective visual vertical, curve-pattern and radiographic variables, and the inter-test association.

*n*	Bucket Test, ρ (*p*; q)	Luminous-Line Test, ρ (*p*; q)	Variable
			**Clinically Identified Curve-Pattern Variables**
57	0.161 (0.233; 0.798)	−0.174 (0.195; 0.798)	Primary curve convexity on right side
57	−0.202 (0.132; 0.721)	−0.083 (0.537; 0.906)	Primary curve convexity on left side
57	−0.014 (0.919; 1.000)	−0.126 (0.348; 0.864)	Primary thoracic curve
57	0.125 (0.355; 0.864)	−0.011 (0.937; 1.000)	Primary thoracolumbar curve
57	−0.205 (0.127; 0.721)	−0.278 (0.036; 0.721)	Primary lumbar curve
57	−0.220 (0.100; 0.721)	−0.169 (0.210; 0.798)	Secondary curve convexity on right side
57	0.085 (0.529; 0.906)	−0.134 (0.322; 0.864)	Secondary curve convexity on left side
56	−0.190 (0.160; 0.787)	−0.006 (0.964; 1.000)	Secondary thoracic curve
56	−0.203 (0.134; 0.721)	−0.324 (0.015; 0.721)	Secondary thoracolumbar curve
56	0.213 (0.115; 0.721)	−0.010 (0.940; 1.000)	Secondary lumbar curve
			**Radiographic variables**
21	0.035 (0.882; 1.000)	−0.234 (0.306; 0.864)	Primary curve magnitude (degrees Cobb)
8	0.000 (1.000; 1.000)	0.007 (0.987; 1.000)	Secondary curve magnitude (degrees Cobb)
16	−0.244 (0.363; 0.864)	0.314 (0.236; 0.798)	Risser sign
57	—	0.493 (<0.001; —)	Luminous-line test vs. Bucket Test

Values are Spearman’s ρ, with the unadjusted *p*-value and false-discovery-rate-adjusted q-value shown in parentheses as ρ (*p*; q). Curve-pattern variables were recorded clinically; Cobb angles and Risser sign were available only in participants who underwent radiography. Statistical significance for exploratory associations was defined as q < 0.05. The inter-test correlation was a separate, prespecified convergent-validity analysis and was not included in the multiple-comparison family.

## Data Availability

The raw data supporting the conclusions of this article will be made available by the authors on request.
